# SHIVA02 Trial: Using Patients as Their Own Controls to Assess Efficacy of Therapies Based on Molecular Profiling in Patients with Refractory Cancer

**DOI:** 10.1158/2767-9764.CRC-26-0225

**Published:** 2026-09-25

**Authors:** Pauline du Rusquec, Célia Dupain, Romain Geiss, Marie-Paule Sablin, Zahra Castel-Ajgal, Philippe Cassier, Antoine Italiano, Edith Borcoman, Jerzy Klijanienko, Vincent Servois, Laurence Raizonville, Fanny Coffin, Julien Romejon, Philippe Hupé, Laëtitia Chanas, Sylvain Dureau, Eleonore Frouin, Odette Mariani, Samantha Antonio, Céline Callens, Samia Melaabi, Julien Masliah-Planchon, Elodie Girard, Nicolas Servant, Maud Kamal, Ivan Bièche, Xavier Paoletti, Christophe Le Tourneau

**Affiliations:** 1 Department of Medical Oncology, Institut Curie, Paris, France.; 2 Department of Drug Development and Innovation (D3i), Institut Curie, Paris, France.; 3 Département de Cancérologie Médicale, Centre Léon Bérard, Lyon, France.; 4 Department of Medical Oncology, Institut Bergonié, Bordeaux, France.; 5 Department of Pathology, Institut Curie, Paris and Saint-Cloud, France.; 6 Department of Radiology, Institut Curie, Paris, France.; 7 Department of Biostatistics, Institut Curie, Paris, France.; 8 Computational Oncology, PSL Research University, Mines Paris Tech, INSERM U1331, Paris, France.; 9 CUBIC Bioinformatics Platform, Mines Paris Tech, Institut Curie, Paris, France.; 10 Data Office, Institut Curie, Paris, France.; 11 Department of Biometrics, Institut Curie, Paris, France.; 12 Université PSL, Paris, France.; 13 Clinical Bioinformatics Unit, Institut Curie, Paris, France.; 14 Department of Pathology, Centre de Ressources Biologiques, Institut Curie, Paris, France.; 15 Department of Genetics, Institut Curie, Paris, France.; 16 National PRecISion Medicine Center in Oncology, Gustave Roussy Cancer Campus, Villejuif, France.; 17 Paris Sciences Lettres (PSL) Research University, Paris, France.; 18 Université Versailles St Quentin and INSERM U1331 STAMPM, Institut Curie, Paris, France.

## Abstract

**Purpose:**

SHIVA02 is a tissue-agnostic precision medicine basket trial assessing a molecularly targeted strategy in patients with refractory cancer using patients as their own controls to assess the efficacy of the strategy.

**Patients and Methods:**

Patients with cancers refractory to standard of care underwent molecular profiling and received standard therapy, followed, if possible, by matched therapy guided by a Molecular Biology Board. Tumor evaluations were performed every 2 months according to RECIST 1.1 on both treatments in order to reliably assess the progression-free survival (PFS) ratio.

**Results:**

Among 263 patients enrolled, molecular profiling was successful in 192 patients (73%), and 34 patients (13%) received matched therapy and were eligible for PFS ratio analyses. The median PFS1 was 2.1 months [95% confidence interval (CI), 1.8–3.6] and the median PFS2 was 2 months (95% CI, 1.8–2.7). A PFS ratio exceeding 1.3 or 1.5 was observed in 11 (32%) and 8 (24%) patients, respectively. Overall survival (OS) was prolonged in the subgroup of patients evaluable for PFS ratio. Prognostic factors such as Eastern Cooperative Oncology Group performance status and Royal Marsden Hospital score significantly influenced OS, highlighting the importance of patient selection in precision oncology trials.

**Conclusions:**

Using a prospective and standardized approach for evaluating PFS on both standard and matched therapies, 32% of patients receiving matched therapy benefited from the approach with a PFS ratio >1.3.

**Significance:**

The SHIVA02 trial evaluated molecularly guided anticancer therapy in patients with refractory cancer. To rigorously assess efficacy in this heterogeneous population, each patient served as their own control using the PFS ratio, comparing two consecutive PFS periods. Unlike previous studies relying on limited or retrospective PFS1 data, SHIVA02 prospectively assessed both periods according to RECIST 1.1 criteria, providing the first trial specifically designed for robust evaluation of the PFS ratio.

## Introduction

The advent of molecular profiling has revolutionized oncology by enabling diagnostic, prognostic, and theranostic applications, leading to the U.S. Food and Drug Administration (FDA) approval of tissue-agnostic therapies that have transformed the treatment landscape. Currently, nine such therapies are FDA approved, targeting specific molecular markers irrespective of the tumor’s origin. These agents are grouped into three classes: targeted therapies against genomic alterations, including NTRK fusions, *BRAF*^V600E^ mutations, and RET fusions; immunotherapies for tumors with high microsatellite instability (MSI), deficient mismatch repair, or a high tumor mutational burden (TMB; ≥ 10 mutations/megabase); and an antibody–drug conjugate for HER2-positive tumors, with HER2 being assessed using immunohistochemistry (1–12). These approvals have primarily emerged from single-arm basket trials that assessed a single drug efficacy across various cancer types that share a specific molecular feature. A key challenge, however, is that the molecular targets for these therapies are sometimes found at low frequencies, leading to prolonged trial durations (1, 2, 5).

To address this, novel trial designs have been used for testing multiple therapies across various cancer types and molecular alterations within a single study. Patients are assigned to a specific agent based on the molecular alteration identified in their tumors. Because of the lack of power to evaluate the efficacy of each drug in every histologically and molecularly defined subgroup of patients, these trials evaluate the overall strategy of the molecularly guided approach, essentially testing the treatment allocation algorithm itself (13). Notable examples of this kind of trial include nonrandomized trials, such as MOSCATO 01 (14) and WINTHER (15), and randomized trials such as SHIVA01 (16), NCI-MPACT (17), IMPACT2 (18), and more recently the ROME trial (19).

However, most of histology-agnostic randomized trials have yielded negative results (16–18). Multiple factors have been proposed to explain these results, reflecting the complexity of interpreting outcomes in heterogeneous trial settings (20–22). First, the theranostic significance of a given molecular alteration can differ substantially between cancer types. Cancer cells employ multiple redundant pathways and adaptive mechanisms that may vary depending on cancer type and prior treatments received. Secondly, it has been observed in these trials that predefined treatment algorithms are short-lived, as they are quickly rendered outdated by advances in molecular oncology and the continuous emergence of new therapies. Consequently, the detection of a clinically relevant actionable alteration in patients randomized to the standard arm may have increased the dropout rate, as observed in the M-PACT study, as both patients and physicians often chose off-trial access to targeted therapies instead (17). Finally, these trials are usually conducted in patients with refractory cancers, who have often undergone multiple lines of therapies and for whom the hope of significantly improving prognosis remains limited. Overall, these outcomes likely reflect the limitations inherent in comparing highly heterogeneous patient populations, in which differences in tumor histology, molecular context, and drug efficacy confound interpretation.

An alternative trial design, intended to address the heterogeneity limitation, has been used in the above-mentioned nonrandomized trials, utilizing patients as their own controls. This design enables a direct comparison of the efficacy of molecularly guided therapy with that of conventional therapy within the same patient, thereby mitigating the heterogeneity inherent to randomized trials that compare two groups of patients. This model was first employed in a pilot study by Von Hoff (23) using for the first time the progression-free survival (PFS) ratio as an efficacy endpoint. The use of this endpoint has been criticized for several reasons, including the retrospective assessment of the PFS1 outside the trial. Indeed, the risk is to assess both PFS with different methods (usually RECIST 1.1 for PFS2 but not for PFS1), thus potentially underestimating PFS1 by defining progressive disease without disease progression per RECIST 1.1 criteria (24). Different disease evaluation timings for PFS1 and PFS2 might also bias these assessments (25).

The SHIVA02 trial (NCT03084757) was designed to also use the PFS ratio as primary endpoint, but with both PFS being assessed during the trial with the same method and the same disease evaluation timings. The objective was to evaluate whether molecularly targeted therapy based on molecular alterations improves outcomes as compared with conventional treatment in patients with any kind of cancer that is refractory to standard of care using each patient as their own control.

## Patients and Methods

### Study overview

This two-step clinical trial was dedicated to patients with previously treated recurrent and/or metastatic cancer. Patients had to undergo a tumor biopsy to obtain a molecular profile of their tumor. Pending analysis of these results, patients received treatment of physician’s choice (TPC; step 1). At disease progression, the results of the molecular analysis were disclosed, and patients could move to step 2 and receive matched therapy based on the results of the molecular analysis.

### Study goal and endpoints

The primary endpoint was the proportion of patients with a PFS ratio exceeding 1.5. PFS was measured from the start date of each treatment to the date of progression according to RECIST 1.1 or the date of death. PFS1 was defined as the PFS during step 1 and PFS2 as the PFS during step 2. Secondary objectives included the evaluation of the proportion of patients with a PFS ratio >1.3, the overall response rate (ORR) of both treatments, overall survival (OS), and safety. Ancillary objectives assessed the concordance and predictive value of ctDNA and fine-needle aspiration (FNA) cytology compared with tumor biopsies.

### Study design

**Figure 1.**
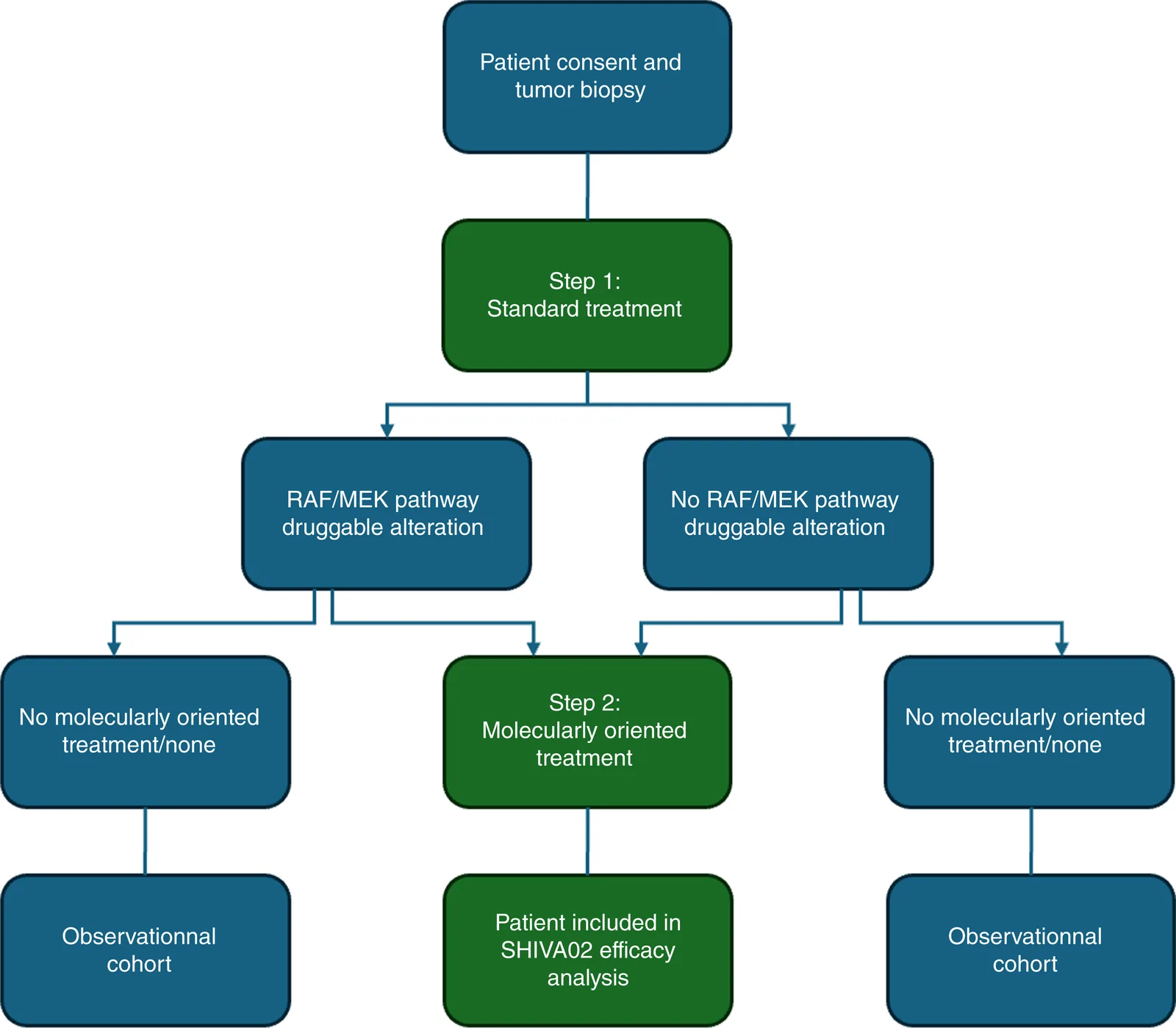
Study design. RAF/MEK, rapidly accelerated fibrosarcoma/mitogen-activated protein kinase kinase.

The SHIVA02 trial was constructed in a two-step manner, detailed in Fig. 1. The study was dedicated to patients with any type of refractory and/or metastatic solid tumor. Initially limited to RAF/MEK pathway alterations based on SHIVA01 results (16), the protocol was later amended to include all patients receiving matched therapy based on molecular profiling during step 1 (V7.0 of March 8, 2022; NCT03084757). This article therefore presents the global SHIVA02 results.

The study was conducted in accordance with the principles of the Declaration of Helsinki. The trial received approval from the Institutional Review Board, the national ethics committee, and the French Agence Nationale de Sécurité du Médicament et des produits de santé.

### Patients consent and eligibility

After signing written informed consent, each patient with documented progressive disease on standard treatment underwent a tumor biopsy to establish the molecular profile of the tumor. The patient was then treated with a standard treatment or simply monitored in the absence of a therapeutic alternative (first step). After step 1 disease progression according to RECIST 1.1, patients received, if available, a matched therapy guided by the molecular profiling of the tumor (step 2), after discussion with Molecular Biology Board (MBB), provided that they met the selection criteria. Otherwise, they entered an observational cohort.

Eligibility criteria:

- Step 1: Eastern Cooperative Oncology Group (ECOG) performance status (PS) 0 to 1 and adequate biological parameters.
- Step 2: Exclusion of patients with central nervous system metastases uncontrolled for >3 months, uncontrolled comorbidity, pregnancy, breastfeeding, or active infections (human immunodeficiency virus and hepatitis B/C).

### Sample collection

Sampling was performed by an interventional radiologist using ultrasound or CT guidance. Biopsy specimens were placed in RPMI medium. Of three core-needle biopsies (CNB), one was fixed in paraffin, one frozen in liquid nitrogen, and one used for quality control. Molecular analysis required >30% tumor cellularity. Translational analyses also used plasma (for ctDNA) and FNA samples collected at inclusion. Only CNB informed diagnostic and therapeutic decisions in the frame of the study.

### Molecular profiling

DNA was extracted from tumor biopsies using phenol:chloroform:isoamyl alcohol (Invitrogen). Samples were quantified by NanoDrop and Qubit, checked on agarose gel, and stored at −20°C. For library preparation, 100 ng DNA was used. Until 2019, an in-house next-generation sequencing (NGS)–based gene panel (99.3 kb and 1,504 amplicons) was used, covering 87 genes using the TruSeq Custom Amplicon Low Input library prep kit (Illumina). From 2019, a new custom NGS panel known as DRAGON (Detection of Relevant Alterations in Genes involved in Oncogenetics by NGS), commercially available as SureSelect CD Curie CGP by Agilent, was used for molecular screening. Both panels included genes used in the SHIVA02 treatment algorithm.

### Bioinformatics

After tumor sequencing, bioinformatics analyses were performed as previously described (26, 27) to detect single-nucleotide variants, indels, and copy number variations for both panels. Standard biomarkers, including MSI status, mutational signatures, TMB, and homologous recombination status, were processed for samples screened with the DRAGON NGS panel.

### MBB review

The MBB was composed of oncologists, pathologists, radiologists, and biologists and met on a weekly basis. The MBB discussed all cases, interpreted and assessed the molecular data to authenticate a druggable alteration, and validated patient eligibility for step 2. If a druggable alteration was validated, the treatment recommendations for step 2 were based on a predefined treatment algorithm for RAF/MEK pathway alterations (Table 1), or on molecular-oriented early-phase clinical trials and evolving market authorizations for other alterations, considering previous treatments received.

**Table 1.** SHIVA02 algorithm.

Molecular alterations	Expected incidence (%)	Class of agents
EGFR mutation/amplification	7	EGFR inhibitor
HER2/HER3 mutation/amplification	7	Pan-HER inhibitor
KIT and PDGFRA/B mutation/amplification	7	KIT and PDGFRA/B inhibitor
BRAF mutation/amplification	42	BRAF inhibitor + MEK inhibitor
K/H/NRAS mutation^a^		
KRAS amplification^a^		
NF1 inactivation		
MAP2K1/MAP2K2 mutation		
MAP2K4 and MAP3K1 inactivation		
RET mutation/amplification	4	RET inhibitor
LCK mutation/amplification	1	LCK inhibitor
ALK MET mutation/amplification	6	ALK and MET inhibitor
CCND1, CDK4–6 amplification, and CDKN2A deletion/mutation	26	CDK4–6 inhibitor

a For nongastrointestinal tumors only.

### Efficacy and tolerance

During both steps, tumor evaluation by CT was performed every 2 months. PFS, OS, and response rates were measured per RECIST 1.1 (28). PFS was evaluated consecutively with standard treatment, received during step 1 (PFS1), and with matched therapy, received during step 2 (PFS2).

OS was measured from the start of step 1 treatment until the date of death from any cause. Patients alive at the end of the SHIVA02 trial were censored at the date of the last visit at site. The Royal Marsden Hospital (RMH) score, combining albumin, lactate dehydrogenase (LDH), and metastatic site count, was used to predict OS. A low RMH score (0–1) has been shown to be associated with better outcomes (29, 30).

### Ancillary studies

To assess the ancillary objectives of the study, the concordance of molecular analysis results obtained by CNB and FNA has already been evaluated and published (26). After ctDNA extraction from plasma, the proportion of patients for whom targetable alterations were detected from CNB and in ctDNA or FNA was assessed, as well as quantitative and qualitative variations in ctDNA molecular alterations (Nedara and colleagues; in preparation).

### Statistical analysis

The PFS ratio was calculated for each patient as the ratio of PFS2/time to progression 1 (TTP1). TTP1 corresponded to the PFS1 of patients from step 1 who proceeded to step 2, thus excluding those who had died or were deemed ineligible for step 2.

Based on the SHIVA01 trial, the median PFS1 of this patient population is expected to be around 2 months (16). The aim is to focus on patients treated with molecularly targeted agents involving the RAF/MEK signaling pathway for which a substantial (although nonstatistically significant) improvement of the median PFS has been reported in the SHIVA01 trial (3.7 months vs. 2 months; *P* = 0.19). In this subset of patients, in the initial protocol, we considered that a PFS2/PFS1 ratio >1.5 in 40% of patients or more would be clinically meaningful. However, we also present here the data using a cutoff of 1.3 to allow comparison with similar studies, which more commonly use this threshold.

Based on a single-stage Fleming design (31) with a minimum success rate of 30% (p0), a first-species risk of 5% (α) and 95% power to detect a success rate of 50%, 62 patients should be included to assess the efficacy of the therapeutic strategy. Of the first 62 patients included in the efficacy analysis, if 25 or more had a PFS ratio >1.5, then the study would be considered positive. Based on data from the SHIVA01 study in terms of the incidence of molecular alterations, the proportion of patients who would leave the study before receiving treatment, and estimated compliance with MBB recommendations, the number of patients to be included in stage 1 was 370 to be able to include 62 patients in the efficacy analysis (step 2).

Kendall correlation coefficients were calculated to assess the association between the PFS ratio and survival outcomes. All statistical analyses were performed using R software and its RStudio environment (version 4.3.1).

## Results

### Patients and study procedures

**Figure 2.**
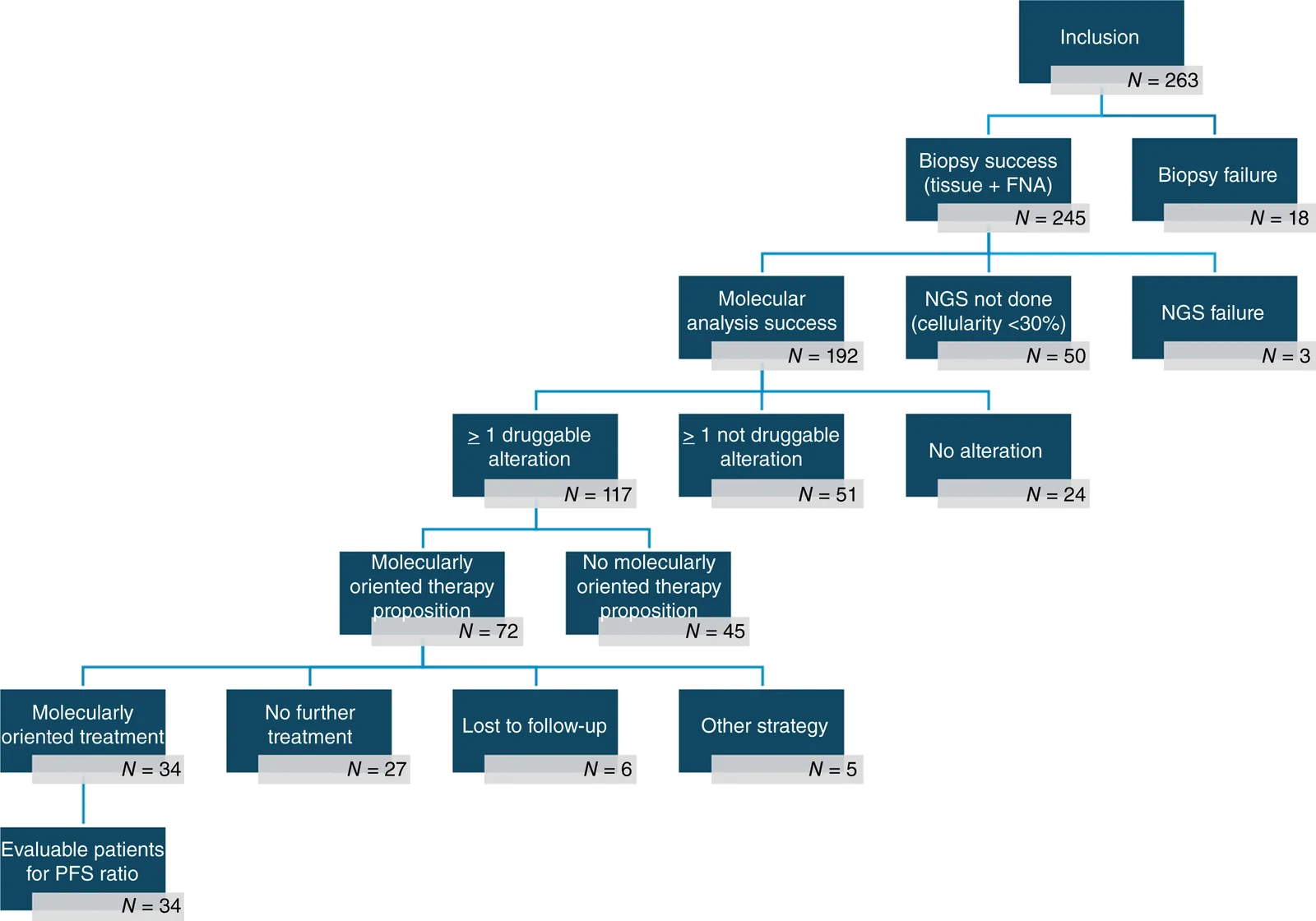
Flow chart of the study. No further treatment = clinical deterioration.

From September 2017 to November 2021, a total of 263 patients were included in the SHIVA02 clinical trial in four French centers (Institut Curie, Paris and Saint Cloud; Centre Léon Bérard, Lyon; and Institut Bergonié, Bordeaux). The flowchart represents the study progress (Fig. 2). A total of 247 patients (94%) underwent a mandatory biopsy of a metastatic site in order to perform the molecular analyses on the most recent sample. Tumor tissue was obtained in 245 (93%) of the 263 patients who consented. Molecular analyses were successful in 192 patients (73%). Thirty-four patients (13%) eventually received matched therapy based on the molecular profile and were evaluable for the PFS ratio analysis. Table 2 summarizes their main characteristics and the overall patient population included in the study. Representativeness of study participants is shown in Supplementary Table S2.

**Table 2.** Patient characteristics.

Characteristic	Patients included in SHIVA02 (*n* = 263)		Patients eligible for PFS ratio (*n* = 34)	
Median age at inclusion (range)	61	(30–89)	60	(39–75)
Gender	​	​	​	​
Male	64	24%	5	15%
Female	199	76%	29	85%
ECOG PS	​	​	​	​
0	96	37%	13	38%
1	143	54%	20	59%
2	3	1%	0	—
Missing	21	8%	1	3%
Year of inclusion	​	​	​	​
2017	33	13%	3	9%
2018	103	39%	12	35%
2019	64	24%	8	24%
2020	47	18%	7	21%
2021	16	6%	4	12%
Primary tumor type	​	​	​	​
Breast	73	28%	11	32%
Gastrointestinal	80	30%	10	29%
Gynecologic	67	25%	9	26%
Head and neck	8	3%	1	3%
Lung and chest	11	4%	2	6%
Sarcoma	9	3%	1	3%
Urological	5	2%	0	—
Missing	6	2%	0	—
Other	4	2%	0	—
Number of metastatic sites	​	​	​	​
Median (range)	3 (0–6)	​	2 (1–5)	​
Missing	17	6%	0	—
Number of prior lines of treatment	​	​	​	​
Median (range)	3 (0–20)	​	3 (1–9)	​
Missing	17	6%	0	—
RMH score	​	​	​	​
Low (0–1)	123	47%	21	62%
High (2–3)	92	35%	8	24%
Missing	48	18%	5	15%

**Figure 3.**
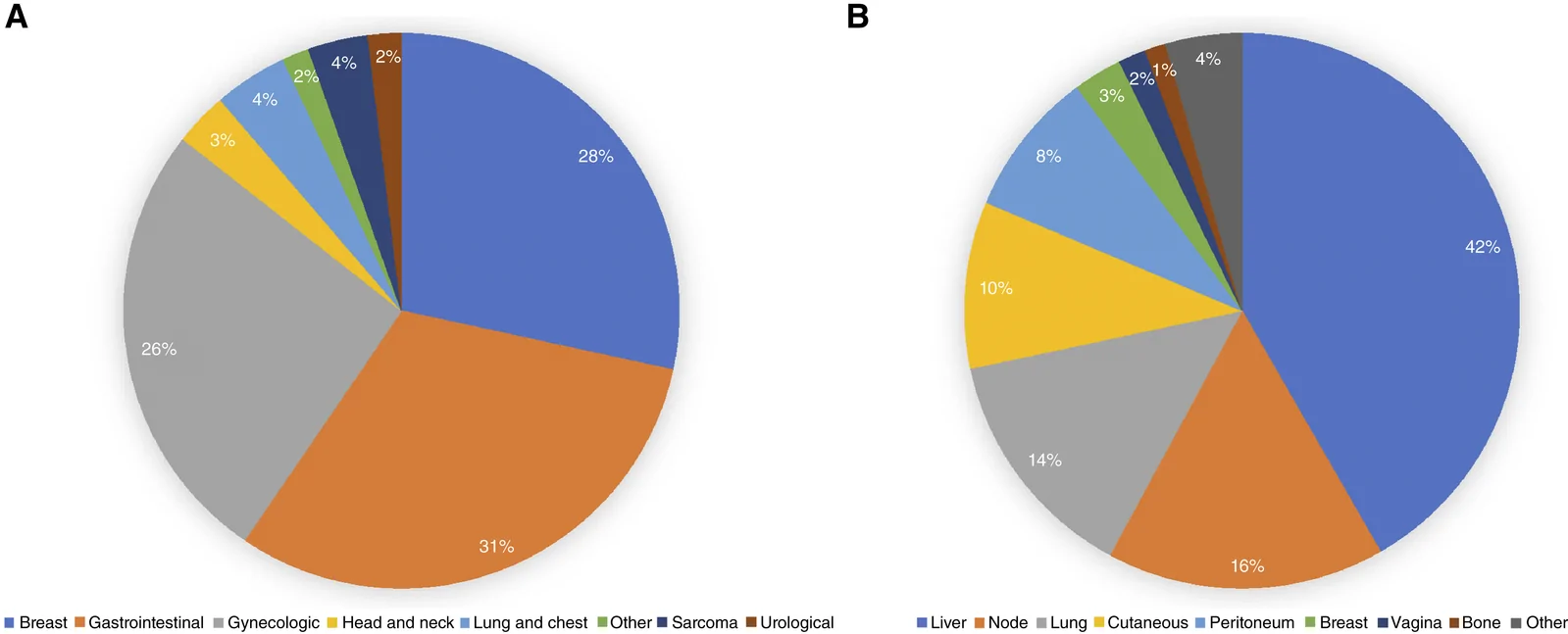
Primary tumor types (**A**) and biopsy sites (**B**).

One biopsy could not be performed given the occurrence of a pneumothorax. Another procedure only involved a cell aspiration but no tumor biopsy. Figure 3 shows the distribution of primary tumor types and biopsy sites. The “other” primary types include one case of histiocytosis, one primary peritoneal tumor, and two carcinomas of unknown primary. The “other” biopsy sites include ovary (*n* = 3), uterus (2), kidney (1), pleura (1), spleen (1), and unknown (3).

### Step 1: standard therapy efficacy

For PFS ratio analyses, four of 34 eligible patients (12%) did not receive any cancer treatment during step 1 and were simply monitored for lack of therapeutic alternative (*n* = 2) or at the patient’s request (*n* = 2). The remaining 30 patients received TPC. The different treatments received during step 1 included paclitaxel (*n* = 4), liposomal doxorubicin (3), gemcitabine (3), capecitabine (2), cyclophosphamide (2), eribulin (2), lonsurf (2), atezolizumab (1), carboplatin (1), folfiri (1), folfox (1), lonsurf + bevacizumab (1), vinorelbine (1), paclitaxel + bevacizumab (1), paclitaxel + carboplatin (1), pemetrexed (1), regorafenib (1), temodal + irinotecan (1), and topotecan (1). Four patients (12%) experienced an objective response during step 1. The disease control rate was 32%. The median PFS1 was 2.1 months [95% confidence interval (CI), 1.8–3.6].

### Step 2: matched therapy efficacy

**Figure 4.**
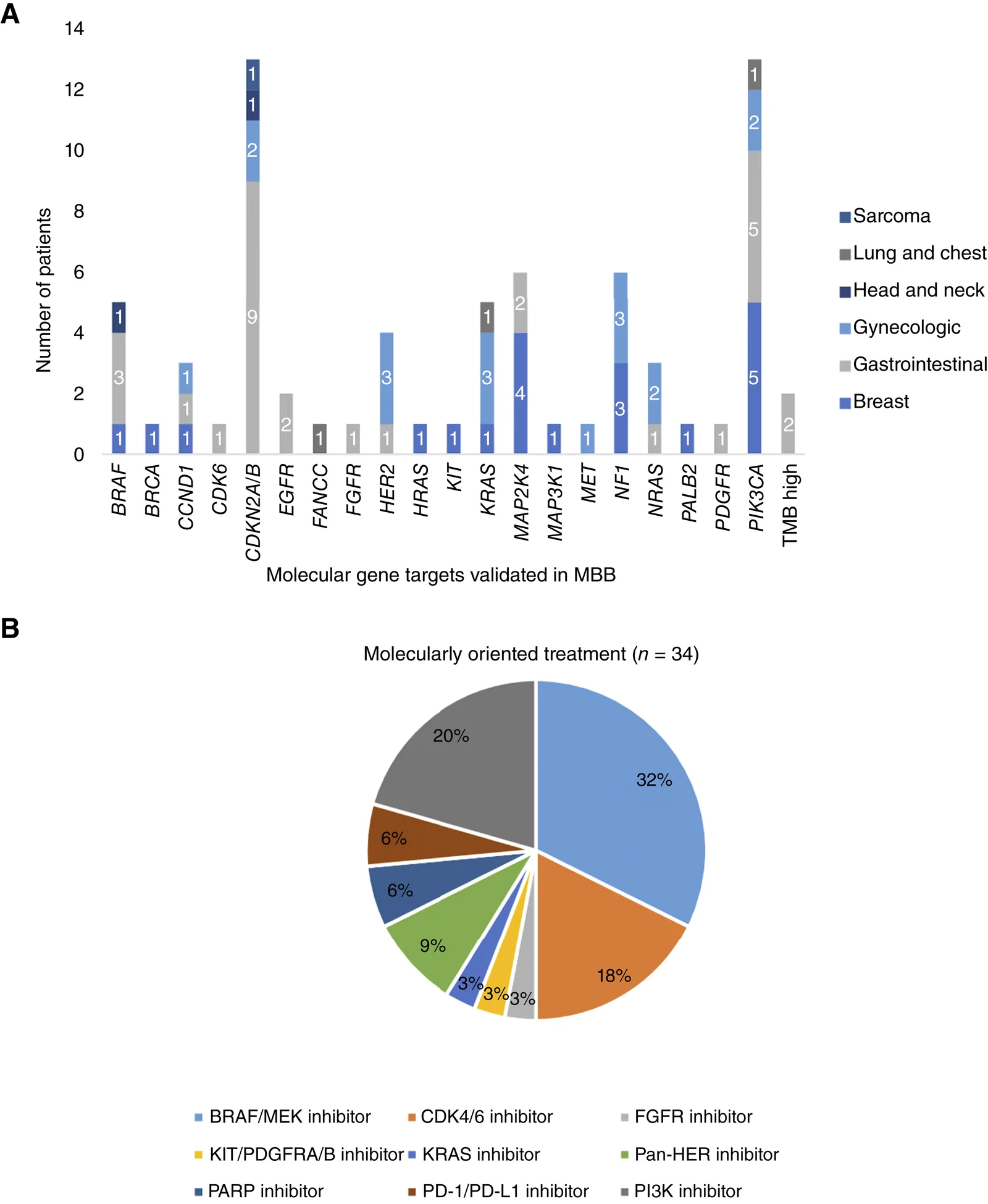
Molecular gene targets validated by MBB for *n* = 72 patients (**A**) and corresponding targeted treatments received for *n* = 34 patients (**B**).

Among the 192 patients with successful molecular analyses, 117 patients (61%) had at least one druggable alteration according to the OncoKB classification (32). Seventy-two patients (38%) were proposed matched therapy, and 34 patients (18%) eventually received it. The actionable mutations validated by the MBB and the treatment decisions are reported by therapeutic classes in Fig. 4.

All 34 patients received matched therapy based on their tumor molecular profiles and included dabrafenib + trametinib (*n* = 10), everolimus (6), palbociclib (5), fulvestrant + alpelisib (2), olaparib (2), abemaciclib (1), durvalumab + tremelimumab (1), erdafitinib (1), imatinib (1), oral PD-L1 inhibitor (1), neratinib (1), sotorasib (1), trastuzumab + neratinib (1), and trastuzumab + pertuzumab (1). One patient (3%) experienced an objective response during step 2. The disease control rate was 24%. The median PFS2 was 2 months (95% CI, 1.8–2.7).

### PFS ratio and survival analysis

Of the 34 patients eligible for PFS ratio analysis, 8 patients (24%) had a PFS ratio exceeding 1.5, and 11 patients (32%) had a PFS ratio exceeding 1.3. Details about those patients are provided in Supplementary Table S1. The median PFS ratio among the 34 patients included in the overall analysis was 0.827. After excluding the four patients who did not receive any treatment during step 1, the median PFS ratio among the remaining 30 patients was 0.812.

**Figure 5.**
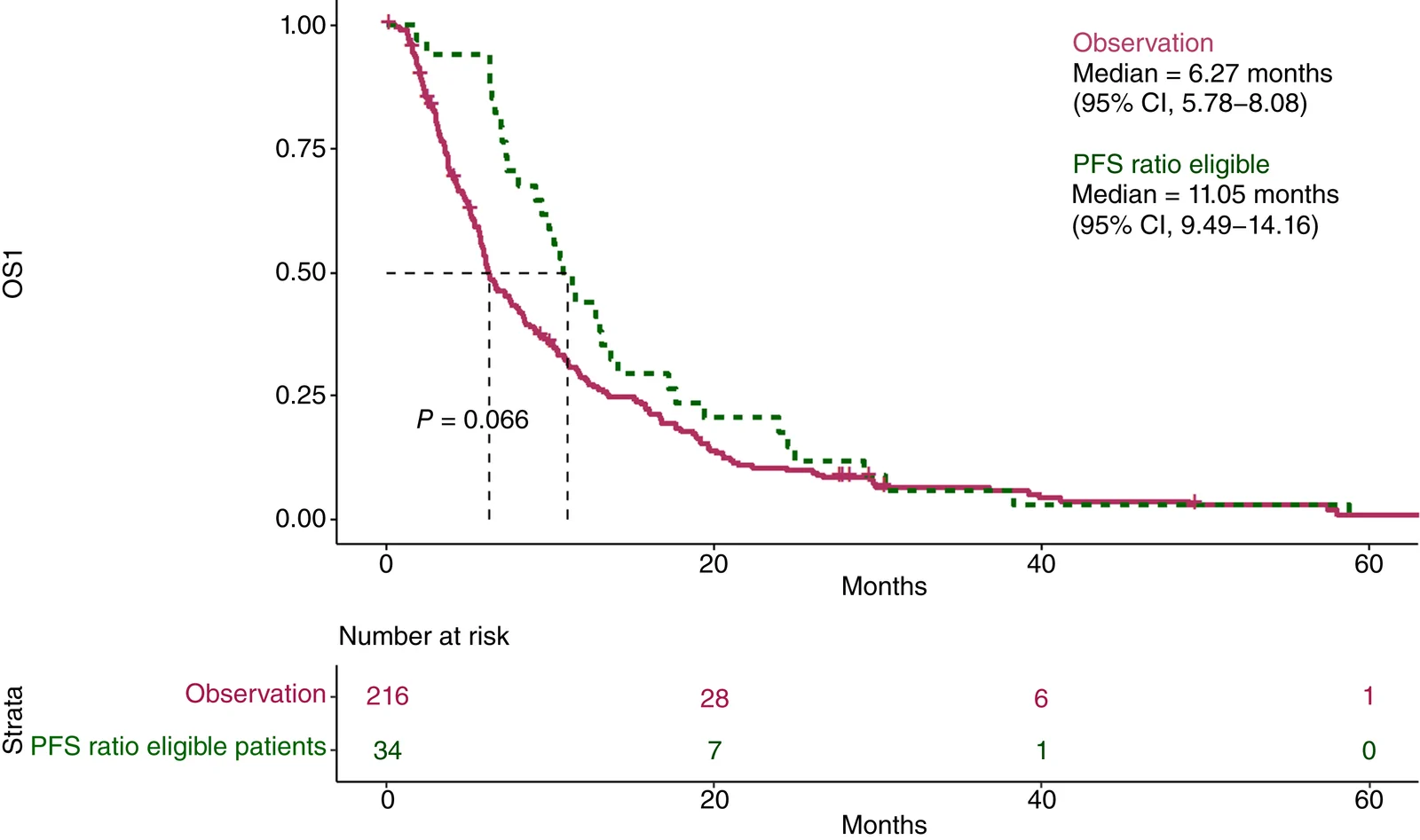
OS according to PFS ratio eligibility. OS was measured since inclusion (OS1).

Survival data were available for 250 of 263 patients who consented to the study. The median OS from inclusion was 7.3 months (95% CI, 6.3–8.8). OS was 11.1 months (95% CI, 9.5–14.2) for the 34 evaluable patients for the PFS ratio assessment and 6.3 months (95% CI, 5.8–8.1) for the remaining patients (Fig. 5). The difference between both groups was not significant (*P* = 0.066).

### Predictive value of PFS and PFS ratio on OS

We report a moderate but significant correlation between the PFS and OS with Kendall correlation coefficients of 0.31 for PFS1 and 0.22 for PFS2. In contrast, there was no significant correlation between the PFS ratio and OS. For OS from inclusion (OS1), the Kendall correlation coefficient was 0.03; for OS measured from the start of step 2 (OS2), it was 0.26; and for OS measured after progression at step 2, it was 0.01.

### Exploratory analyses of prognostic factors

**Figure 6.**
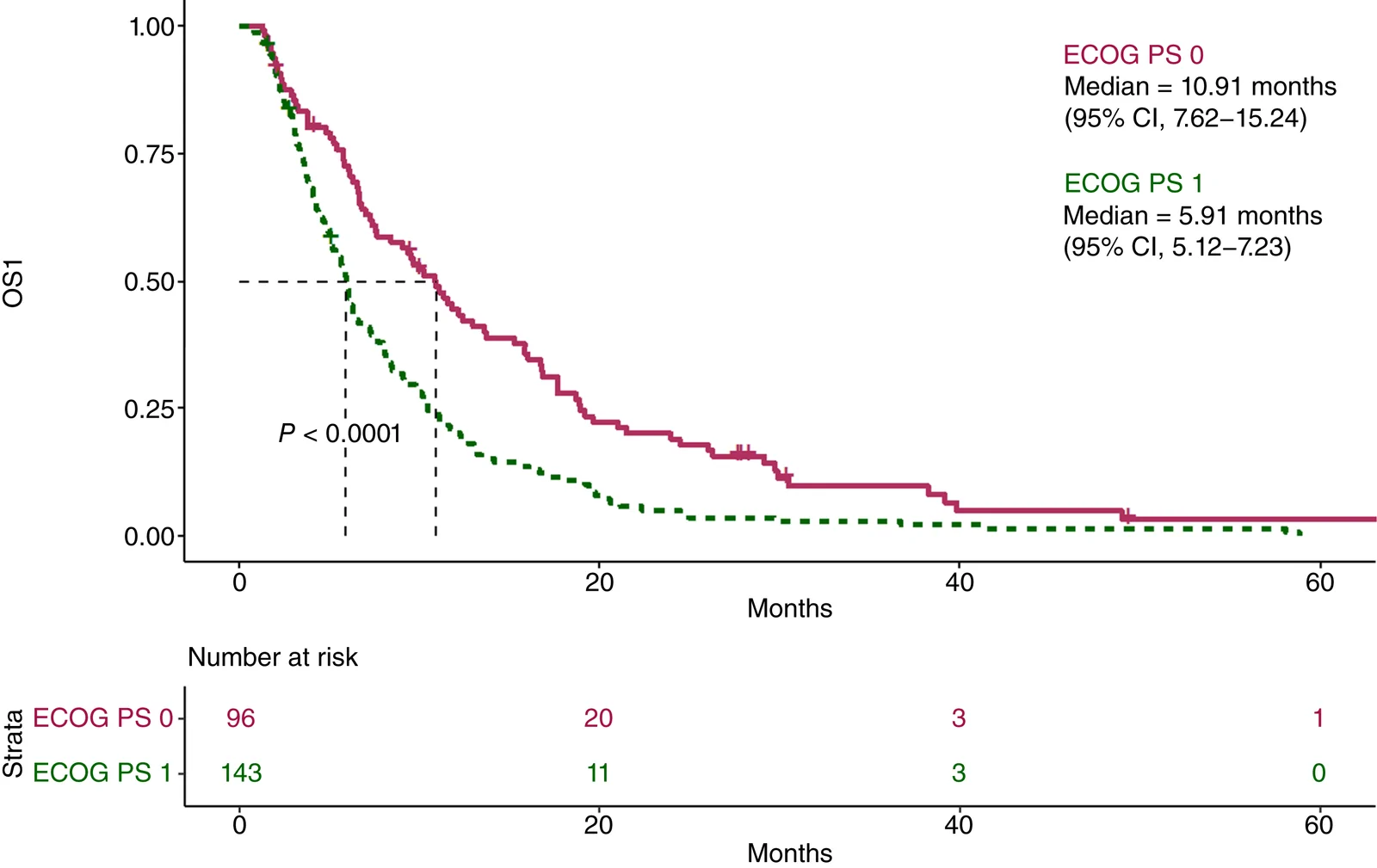
OS according to ECOG PS. OS was measured since inclusion (OS1).

To better understand the efficacy of the strategy proposed in the SHIVA02 trial, we correlated key prognostic factors with the outcomes, including ECOG PS, the number of prior treatment lines in the recurrent and/or metastatic setting, and the RMH score. Due to the limited number of patients who shifted to step 2, these analyses were conducted in the overall population. The median OS was 10.9 months (95% CI, 7.6–15.2) for patients with ECOG PS 0 versus 5.9 months (95% CI, 5.1–7.2) for patients with ECOG PS 1 (*P* < 0.0001; Fig. 6). The median OS was 7.6 months (95% CI, 6.3–9.9) in patients who received three or less prior lines of therapy versus 6.4 months (95% CI, 5.8–8.5) in patients who had received more than three lines of prior therapy (*P* = 0.057; Supplementary Fig. S1). The median OS was prolonged in patients with a low RMH score [9.3 months (95% CI, 7.2–11.5) vs. 5.9 months (95% CI, 4.7–6.6); *P* < 0.0001; Supplementary Fig. S2].

## Discussion

Using a rigorous methodology in which both PFS1 and PFS2 were assessed according to RECIST 1.1 every 2 months, we found in SHIVA02 a similar proportion of patients with a PFS ratio exceeding 1.3 as compared with past trials such as MOSCATO 01 and WINTHER (14, 15). However, assessing both PFS during the trial led to a high proportion of patients who were no longer eligible to receive matched therapy at disease progression following physician’s choice of therapy.

The primary endpoint in SHIVA02 was the proportion of patients with a PFS ratio greater than 1.5 as outlined in the initial protocol. PFS was measured from the start date of each treatment to the date of disease progression according to RECIST 1.1 or the date of death. In the literature, the cutoff for the PFS ratio ranged from 1.2 (33) to 1.5 (15), although most studies use 1.3 (14, 34–42).

Strictly speaking, the PFS ratio is the ratio of the PFS obtained to the prior TTP. In similar studies, TTP1 was generally collected retrospectively. In addition, the timing of disease assessments was often unclear. We also know that longer intervals between two assessments are a source of PFS inflation (25). Therefore, the accuracy of the PFS ratio measurement is a crucial issue. In this pancancer basket trial setting, the PFS ratio was used as a within-patient benchmark, allowing each patient to serve as their own control; however, its interpretation is limited by selection bias because of the low proportion of patients reaching the second step and by its status as a nonvalidated surrogate endpoint. SHIVA02 was designed to use the most rigorous PFS ratio evaluation method possible. First, both therapeutic sequences were part of the clinical trial (standard therapy to assess TTP1 followed by molecularly oriented therapy to assess PFS2). Consequently, in our study, TTP1 and PFS2 were measured prospectively. Finally, the RECIST 1.1 assessment schedule was identical during both steps of the SHIVA02 study, with CT scans performed every 2 months, avoiding analysis time bias.

To potentially improve the inclusion-to-efficacy patient ratio, SHIVA02 focused on RAF/MEK pathway abnormalities identified through molecular profiling. However, this static approach, which did not account for market evolution and targeted therapies, would have allowed only 19 patients to be treated and evaluated according to the initial protocol. To take a more dynamic approach, SHIVA02 was amended (v7.0 of March 8, 2022; NCT03084757) to include in its final analysis all patients who actually received a treatment guided by their molecular profile obtained during the study. Therefore, 15 additional patients have been included in the efficacy analysis. Although this protocol amendment was a limitation, it also became a strength, as it enabled a more robust evaluation of the study’s main objective, as initially formulated.

Our study was terminated prematurely, primarily because of the low proportion of patients reaching step 2, which made it impossible to achieve the target number of patients evaluable for the PFS ratio. Consequently, the study did not reach the planned number of evaluable patients required by the initial Fleming design, reducing statistical power and limiting our ability to detect the anticipated treatment effect. Therefore, the efficacy results should be interpreted with caution. Furthermore, although the primary analysis was based on the protocol-defined PFS ratio threshold of 1.5, we also report outcomes using a threshold of 1.3 to facilitate comparison with previously published precision oncology trials.

There were several reasons for the lack of patients reaching step 2. In nearly 50% of cases, patients were unable to proceed to step 2 because of a deterioration in their general clinical condition, rendering them ineligible for a new line of treatment. Our results suggest that earlier molecular profiling in the disease course, rather than selection based on patient fitness, could improve the feasibility of delivering matched therapies by increasing the proportion of patients remaining eligible for subsequent treatment at progression.

Our study also allowed for the reassessment of the prognostic value of several factors in this population of patients with poor-prognosis refractory cancers, as previously reported (29, 43). Overall, the prognostic factors that best predicted OS, independently of treatment, included ECOG PS, LDH level, albumin level, and the RMH score, which itself includes LDH level, albumin level, and the number of metastatic sites. Beyond therapeutic considerations, our study confirms that taking these factors into account is fundamental in guiding decisions about pursuing further cancer-specific treatments, as they are associated with prognosis.

The ORR observed during step 2 in our study was low (3%), which is in line with the literature (33–35, 39–42). Furthermore, the median durations of PFS1 (obtained with standard treatment) and PFS2 (obtained with targeted treatment) were similar (2.1 and 2 months, respectively), confirming the refractory nature of patients’ cancer included in our study. PFS1 was provided in only three other precision medicine trials and ranged from 2 to 4.8 months, with a median of 2.27 months (33, 34, 42). PFS2 ranged from 2 to 6.1 months with a median of 2.7 months in the literature (15, 33, 34, 37–39, 41, 42). These data underscore the advanced nature of the disease in the study population and the significant therapeutic challenge this patient population represents.

Given the limited numbers of patients included in the final analysis of SHIVA02, we chose the Kendall rank correlation method, which is better suited for small sample sizes, to measure the potential correlation between the PFS ratio and survival. We found no significant correlation between the PFS ratio and OS. In contrast, there was a moderate but significant correlation between PFS1 and OS and between PFS2 and OS. Despite a limited efficacy, both lines of cancer therapy were independently predictive of OS, whereas the effect of the therapeutic sequence, embraced by the PFS ratio, did not predict OS. This could be explained, on the one hand, by the heterogeneity of the population but also by the treatments potentially received after step 2.

The median OS measured from inclusion, of all patients included in SHIVA02, was 7.3 months. The median OS of patients evaluable for the PFS ratio was 11.1 months. In other precision medicine trials, the OS of patients treated and evaluable for the PFS ratio was highly variable, ranging from 5 to 12 months, except for the 25 months described in Miller and colleagues study (14, 15, 33–35, 39–42). The median OS of patients who were not evaluable for the PFS ratio was, not surprisingly, shorter (6.3 months). Although as previously highlighted, the use of the PFS ratio as a trial endpoint seems to select a population with a better prognosis than the overall patient population (44, 45). Despite the modest response rate and PFS observed in our study, the prolonged OS demonstrated in the cohort of patients treated at step 2 compared with the observational cohort alone justifies, even for a patient in a later line of therapy, screening them for a potential targeted treatment. Using more flexible algorithms in a fitter patient population could enhance the utility of the PFS ratio as an endpoint for assessing the effect of a therapeutic sequence.

In summary, SHIVA02 with its robust methodology confirms the results of other precision medicine trials that used the PFS ratio even though PFS1 was retrospectively assessed. The downside of having patients receiving both unmatched and matched therapies during the trial is that a high proportion of patients became clinically unfit to receive the matched therapy.

## Supplementary Material

Supplementary Figure S1 Overall survival according to the number of prior lines of systemic treatment

Supplementary Table S1 Detailed characteristics of patients evaluable for the PFS ratio

Supplementary Figure S2 Overall survival according to the RMH score

Supplementary Table S2 Representativeness of study participants

## Data Availability

Original files and raw data files from the SHIVA02 study will be made available from the corresponding authors upon reasonable request. The data are not publicly available because of information that could compromise the privacy of the research participants.
